# Ultrasound‐Assisted Extraction of Bioactive Compounds From Black Pine ( *Pinus nigra* ) Bark: Optimization and Evaluation of Their In Vitro Bioactivities

**DOI:** 10.1002/fsn3.70224

**Published:** 2025-05-15

**Authors:** Kubra Feyza Erol, Gozde Kutlu, Necattin Cihat Icyer, Fatih Tornuk

**Affiliations:** ^1^ University of Health Sciences, Hamidiye Faculty of Health Sciences, Department of Nutrition and Dietetics Istanbul Türkiye; ^2^ Ankara Medipol University, Faculty of Fine Arts, Design and Architecture, Department of Gastronomy and Culinary Arts Ankara Türkiye; ^3^ Department of Food Engineering, Faculty of Engineering and Architecture Muş Alparslan University Muş Türkiye; ^4^ Sivas Cumhuriyet University, Faculty of Health Sciences, Department of Nutrition and Dietetics Sivas Türkiye

**Keywords:** anticancer activity, antioxidant activity, optimization, *Pinus nigra* bark, ultrasound‐assisted extraction

## Abstract

In the present study, the effects of extraction temperature, extraction time, and ultrasonic power on extraction yield, ferric reducing antioxidant power (FRAP), total phenolic content (TPC), ABTS activities, as well as total condensed tannin (TCT) content of black pine bark extracts (PBE) were specified using the Box–Behnken experimental design. The current study also shed light on their potential anticancer, antimicrobial, antidiabetic, and anticholinesterase activities at the estimated optimal conditions. The estimated optimal conditions to achieve maximum TPC (128.00 mg GAE [gallic acid equivalent]/g of dried bark extract [dbe]), TCT (22.08 mg CE (catechin equivalents)/g of dbe), FRAP (649.49 mg TEAC (trolox equivalent antioxidant capacity)/g of dbe) and ABTS activities (802.04 mg TEAC/g of dbe) were as follows: extraction temperature of 32.52°C, extraction time of 7.43 min, and ultrasonic power of 110.68 W for PBE. Additionally, phenolic and organic compounds most commonly found in PBE were succinic acid (16.35 mg/100 g), gentisic acid (7.58 mg/100 g), and oxalic acid (6.81 mg/100 g), respectively, based on the LC–MS/MS analysis. Furthermore, PBE exhibited weak cytotoxicity across all tested cells (Caco‐2, MIA PaCa‐2, and HEK‐293); however, its cytotoxic effects were slightly less pronounced in the healthy HEK‐293 cells compared to the Caco‐2 and MIA PaCa‐2 cancer cells. Accordingly, PBE showed stronger antidiabetic activity than acarbose, the reference antidiabetic drug, with half‐maximal inhibitory concentration (IC_50_) values of 0.38 mg/mL and 0.46 mg/mL against α‐glucosidase and α‐amylase, respectively, compared to acarbose's IC_50_ values of 0.72 mg/mL and 0.66 mg/mL. At 2 mg/mL, PBE showed moderate anticholinesterase activity, inhibiting acetylcholinesterase (AChE) by 57.26% and butylcholinesterase (BChE) by 48.35%, compared to the stronger effects of galantamine hydrobromide, which inhibited AChE by 88.57% and BChE by 85.72%. These findings highlighted the potential of PBE as a natural source of bioactive compounds with diverse biological activities, including antioxidant, antidiabetic, anticancer, and anticholinesterase properties.

## Graphical abstract

Black pine bark extract holds significant potential as a versatile bioactive ingredient for the development of functional foods. Its antioxidant, antidiabetic, and health promoting properties make it valuable for enhancing the nutritional and functional quality of food products. Its incorporation into foods can also enhance their nutritional properties while extending their shelf life.

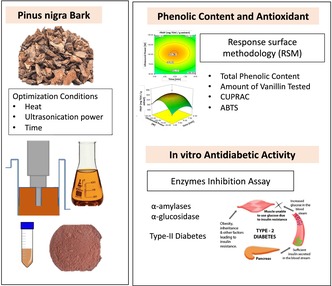

## 1 Introduction

In all over the world, there has been a growing interest to natural phytochemicals due to their desirable health benefits, especially on chronic diseases and their economical importance; hence, the determination of extraction and purification stages from plant materials is highly significant to benefit from the advantages of phytochemicals (Erol and Kutlu 2024a, 2024b; Liu et al. 2021). It was stated that the utilization of conventional methods (e.g., thin‐layer chromatography, decoction, maceration, and percolation) could bring a lot of limitations and problems in terms of efficiency and process (Mondal et al. 2024). On the other hand, nonconventional novel extraction strategies (e.g., ultrasound‐assisted extraction, microwave‐assisted extraction, supercritical fluid extraction, pressurized liquid extraction) can bring many advantages such as faster extraction, higher yield, lower energy consumption, and lower negative environmental effects (Shrivastav et al. 2024). However, during extraction, process parameters should be optimized so as to achieve the highest extraction yield and purity of the phytochemicals (Belwal et al. 2020). Although many operational parameters can affect the bioactive compounds and yield during the ultrasonic extraction application, the extraction time, extraction temperature, and ultrasonic power were selected as the most critical process parameters in this research, and 80% methanol–water was used as a solvent. Moreover, the range of the extraction temperature was selected considering the instability problems of several polyphenols because of the exposure to elevated extraction temperatures for prolonged periods (Deng et al. 2018). In a study, it was reported that rising the extraction time beyond a certain point did not elevate the rate of extraction yield related to the “regular regime” phenomenon (Morsli et al. 2021). Additionally, ultrasonication makes a great contribution to increase the extraction yield thanks to the transmission of ultrasound pressure waves through the solvent and the emergence of cavitation effect (Rao et al. 2021). Also, ultrasonication exhibits mechanical, cavitation, and thermal effects provoking cell wall disruption and diminishing of particle size; as a result of these effects, mass transfer across cell membranes can be increased (Makris 2021). In light of all the facts mentioned above, it is aimed to select adequate extraction conditions with the Box–Behnken design (BBD) to obtain more efficient extraction and bioactive compound results. BBD has been commonly used for the optimization of processes (Al‐Amoudi et al. 2019; Erol et al. 2023; Kutlu, Bozkurt, et al. 2020; Kutlu, Baslar, et al. 2024; Kutlu, Akman, et al. 2024) aiming for the enhancement of phytochemical content and quality of extracts. Polar solvents like methanol or aqueous solutions containing ethanol are often utilized to recover phenolic constituents from natural raw materials (Chemat et al. 2019).

*Pinus nigra* , also known as Austrian pine or black pine, belongs to the genus *Pinaceae*, which includes 200 species, mostly distributed over a vast range, from the Northern Mediterranean and Southwestern Europe to Asia Minor, Crimea, and North Africa (Morocco and Algeria). *Pinus nigra* bark generates almost 10%–24% of total pine volume. Outstanding features of pine bark, such as its high content of bioactive compounds, eco‐friendly by‐products, low cost, and easy availability, has enabled it to be included in a number of studies on food and pharmacological applications (Milić et al. 2021; Ghoreishi et al. 2016).

The increasing understanding of the chemical composition, biological properties, antiproliferative activities, and antimicrobial effects of *Pinus nigra* bark, coupled with advancements in analytical tools such as GC–MS for detecting volatile and polyphenolic substances, highlights its potential as a promising natural ingredient for functional food formulations (Nisca et al. 2021; Ghoreishi et al. 2016; Bianchi et al. 2018; Romani et al. 2006; Ulukanli et al. 2014; Milić et al. 2021; Hafızoğlu et al. 2002; Ince et al. 2009; Touriño et al. 2005). However, it is crucial to note that previous studies have reported relatively low bioactive compound levels of *Pinus nigra* bark, underscoring the importance of optimizing process conditions to enhance bioactive properties and extraction yield. As far as our literature search shows, this is the first comprehensive evaluation and optimization of the bioactive properties of *Pinus nigra* bark extracts, aiming to expand the understanding of its potential applications in food related fields. Overall, the objectives of this study were (i) to determine the effects of three critical factors—extraction time, extraction temperature, and ultrasonic power—on five responses (extraction yield, TPC, TCT, ABTS, and FRAP activity) and optimize the extraction conditions using a Box–Behnken design (BBD) to support process control; (ii) to analyze the pine bark extracts (PBE) composition using liquid chromatography–mass spectrometry (LC–MS/MS); and (iii) to investigate the in vitro biological activities of PBE under optimized conditions, including anticancer, antimicrobial, antidiabetic, and anti‐Alzheimer's activities, along with its effects on the viability of cancer and healthy cell lines.

## 2 Materials and Methods

### 2.1 Preparation of Bark Samples

*Pinus nigra* bark samples were collected from areas evenly distributed throughout the forest region in Isparta, Türkiye. This was carried out on 1 December 2019 according to the TAPPI T 257 cm‐02 standard, and bark samples were quickly sent to the laboratory for analysis as previously reported by Erol et al. (2023) and Erol, Kutlu, et al. (2024). The obtained bark samples was initially freeze‐dried using a lyophiliser (Martin Christ, Beta 1‐8 LSC plus, Osterode am Harz, Germany), followed by grinding with a Wiley mill to a particle size smaller than 1 mm to ensure consistency during the optimization process. Then it was stored in air‐proof plastic bags until extraction process.

### 2.2 Experimental Design and Optimization for PBE

A Box Bhenken Design (BBD), created by Design Expert software (Version 7.0.0, Stat‐Ease Corporation, Minneapolis, MN, USA), was used for optimization of the experimental extraction conditions. The BBD was utilized to evaluate the influences of three independent variables—extraction temperature (*X*_1_, 20°C–40°C), extraction time (*X*_2_, 3–9 min), and ultrasonic power (*X*_3_, 50–150 W)—on the yield and selected bioactive properties (TPC, TCT, ABTS and FRAP activities) for PBE, as shown in Table 1. The levels of process variables were determined according to preliminary experiments and a review of relevant literature. The experimental design consisted of 17 randomized trials with three replications and three factors, as presented in both coded and uncoded forms in Table 1. The independent variables were coded as follows: 0 for the medium level, 1 for the high level, and − 1 for the low level. The extraction from the bark samples was carried out using 80% aquous methanol, following the method described by Erol et al. (2023). For this process, a total of 10 g of lyophilized bark sample was mixed with the solvent at a 1:10 (w/v) ratio and subjected to ultrasonic extraction using an ultrasonic probe. The resulting bark extract was filtered through Whatman No. 1 filter paper, and the solvent was subsequently removed using a rotary evaporator. The final supernatant was then kept at 4°C until further analysis.

**TABLE 1:** Coded and uncoded values for the Box–Behnken design for black pine ( *Pinus nigra* ) bark and the corresponding mean values for extraction yield and bioactive properties.

Run order	Coded values			Actual values			Extraction yield (%)	Bioactive properties			
	*X*_1_	*X*_2_	*X*_3_	Temperature (°C)	Time (min)	Ultrasonic power (Watt)		TPC (mg GAE/g of dbe)	TCT (mg CE/g of dbe)	FRAP activity (mg TEAC/g of dbe)	ABTS activity (mg TEAC/g of dbe)
1	−1	1	0	20	9	100	7.59 ± 0.09	78.84 ± 0.18	15.93 ± 0.06	550.36 ± 1.54	660.75 ± 6.79
2	1	1	0	40	9	100	13.57 ± 0.17	120.46 ± 0.54	21.58 ± 0.07	600.56 ± 2.63	760.35 ± 7.38
3	0	0	0	30	6	100	14.39 ± 0.23	125.71 ± 0.42	20.14 ± 0.10	640.78 ± 4.31	710.16 ± 13.07
4	0	1	1	30	9	150	11.46 ± 0.18	102.36 ± 0.35	22.35 ± 0.08	578.34 ± 7.92	810.42 ± 11.07
5	0	0	0	30	6	100	15.03 ± 0.16	126.43 ± 0.33	21.78 ± 0.07	650.78 ± 6.24	790.72 ± 10.99
6	−1	−1	0	20	3	100	7.03 ± 0.057	70.37 ± 0.21	12.49 ± 0.11	571.03 ± 4.36	580.28 ± 2.63
7	0	0	0	30	6	100	12.35 ± 0.16	126.85 ± 0.33	22.16 ± 0.10	650.35 ± 4.20	830.46 ± 10.02
8	1	0	−1	40	6	50	12.24 ± 0.21	100.78 ± 0.37	17.94 ± 0.09	566.13 ± 6.22	650.25 ± 9.52
9	−1	0	1	20	6	150	7.56 ± 0.13	74.62 ± 0.13	15.38 ± 0.05	540.37 ± 7.16	601.37 ± 3.18
10	0	−1	1	30	3	150	9.57 ± 0.11	90.26 ± 0.16	17.85 ± 0.11	585.67 ± 4.54	729.45 ± 11.71
11	1	0	1	40	6	150	9.86 ± 0.10	99.28 ± 0.20	17.07 ± 0.09	560.46 ± 9.61	699.43 ± 6.21
12	−1	0	−1	20	6	50	10.25 ± 0.18	62.76 ± 0.11	13.52 ± 0.09	520.76 ± 7.97	540.81 ± 2.39
13	0	1	−1	30	9	50	9.51 ± 0.17	86.45 ± 0.08	16.11 ± 0.06	550.75 ± 3.22	700.37 ± 6.92
14	0	0	0	30	6	100	13.45 ± 0.23	127.65 ± 0.23	21.98 ± 0.04	680.75 ± 12.19	796.34 ± 8.61
15	1	−1	0	40	3	100	9.11 ± 0.17	100.58 ± 0.18	18.23 ± 0.03	583.78 ± 4.54	700.16 ± 9.45
16	0	−1	−1	30	3	50	7.72 ± 0.11	90.63 ± 0.11	17.18 ± 0.04	560.46 ± 4.36	640.75 ± 3.39
17	0	0	0	30	6	100	14.63 ± 0.14	120.34 ± 0.21	19.35 ± 0.05	660.48 ± 7.14	733.84 ± 5.02

*Note:* *X*_1_, *X*_2_ and *X*_3_ were the model parameters; extraction temperature, extraction time and ultrasonic power, respectively.

Abbreviations: CE, catechin equivalents; dbe, dried bark extract; FRAP, ferric reducing antioxidant power; GAE, gallic acid equivalents; TCT, total condensed tannin; TEAC, trolox equivalents antioxidant capacity; TPC, total phenolic content.

### 2.3 Extraction Yield

The extraction yield was determined gravimetrically by calculating the percent of the weights of the extract and the bark sample at each experimental point, following the method described by Santos et al. (2012).

### 2.4 Measurement of TPC, TCT, FRAP, and ABTS Activities

The total phenolic content (TPC) of the PBE was quantified using the Folin–Ciocalteu reagent, with gallic acid as the standard, as outlined by Yasar et al. (2022); Demirkan et al. (2024); Süren et al. (2024); Erol et al. (2024b). The total condensed tannin (TCT) values were measured following the spectrophotometric method described by Makkar et al. (1995). The ferric reducing antioxidant power (FRAP) assay was conducted according to the procedure detailed by Çelik et al. (2010). The ABTS assay was performed based on the methodology reported by Apak et al. (2004).

### 2.5 Liquid Chromatography–Tandem Mass Spectrometry (LC–MS/MS) Analysis

The identification and quantification of phenolic compounds in the PBE were performed by a chromatographic assay previously reported by Pelvan et al. (2018) by using LC–MS/MS (AB SCIEX API 4000 QTrap, Framingham, MA, USA) and Q‐OT‐MS (Q‐exactive hybrid quadrupole‐orbitrap mass spectrometer, Thermo Scientific, Bremen, Germany). Q‐OT‐MS was employed in order to specify the phenolic profiles of PBE. A column (ZORBAX Eclipse Plus Phenyl–hexyl 4.6 × 150 mm 3.5 μm, Agilent Technologies, Santa Clara, CA, United States) was utilized in order to separate analytes. Using methanol: acetonitrile (65:35, v/v) as mobile phase B and formic acid‐water (0.1:99.9, v/v) as mobile phase A, the gradient elution was carried out. The multistep gradient that follows was used: 0–1 min, 5% B; 1–30 min, 5%–100% B; 30–31 min, 5% B; 31–40 min, 5% B. Additionally, the following were the operation parameters: Gas 1 and gas 2 temperatures are 50°C, the source temperature is 550°C, and the ion spray voltage is −4500 V (Erol, Kutlu, et al. 2024).

### 2.6 Determination of Minimum Inhibitory Concentration (MIC)

The antibacterial efficacy of the PBE was assessed by determination of minimum inhibition concentration for each test bacterial strain, in accordance with the protocol outlined by Erol et al. (2023). For this purpose, 2‐fold PBE solutions were prepared at concentrations varying from 5 mg/mL to 0.078 mg/mL (5, 2.5, 1.25, 0.625, 0.312, 0.156, and 0.078 mg/mL) using Nutrient Broth and autoclaved. Cultures of *Bacillus cereus* FMC–19, *Staphylococcus aureus* ATCC25923 (+), *Escherichia coli* O157:H7 ATCC33150 (−), *Salmonella enterica* subsp. *enterica* serovar Typhimurium ATCC14028 (−), *Listeria monocytogenes* ATCC19118 (+) were activated in Nutrient Broth at 37°C for 24 h for two times, and 50 μL of the fresh cultures were inoculated to the PBE solutions. Then, the tubes were incubated for 24 h at 37°C. As a negative control, mediums without the test bacteria were also incubated under the same conditions. At the end of the incubation, the lowest PBE concentration at which there was no bacterial growth in the test tubes that completed the incubation period was determined as minimum inhibitory concentration (MIC, μL/mL).

### 2.7 Anticancer Activity Assay

#### 2.7.1 Cell Culture

The in vitro cytotoxic effects of PBE were tested on two different cancer cell lines and one healthy cell line. The selected cancer cell lines were human colorectal adenocarcinoma (Caco‐2, ATCC #HTB‐37) and human pancreatic adenocarcinoma (MIA PaCa‐2, ATCC CRL − 1420), while the healthy cell line was human embryonic kidney cells (HEK‐293, ATCC CRL‐1573). Before cultivation, the cells were cryopreserved in liquid nitrogen. Subsequently, the cells were cultured in a humidified environment at 37°C using DMEM (Pan Biotech, Aidenbach, Germany) medium supplemented with 10% fetal bovine serum (Gibco, Waltham, MA, USA), 1% (v/v) L‐glutamine, and 1% penicillin/streptomycin. The cultures were maintained in 25 cm^2^ plastic flasks under a 5% CO_2_ and 95% air atmosphere (Erol et al. 2023).

#### 2.7.2 Cell Viability Test (XTT Assay)

The in vitro cytotoxicity tests were conducted using XTT method, following the protocol described by Bozkurt et al. (2021) and Kutlu, Akman, et al. (2024). Cells were prepared as outlined above and counted using a hemocytometer (Marienfeld, GmbH, Germany). They were then seeded into 96‐well microplates at a concentration of 10^4^ cells per well and incubated at 37°C for 24 h. After incubation, PBE at different concentrations was added to the wells, and the plates were incubated again at 37°C in a 5% CO_2_ atmosphere for 24 h. Then the medium in each well was removed, and 100 μL of XTT solution (2,3‐bis‐(2‐methoxy‐4‐nitro‐5‐sulfophenyl)‐2H‐tetrazolium‐5‐carboxanilide; 0.5 mg/mL) was added. The plates were then incubated at 37°C for an additional 3 h. At the end of the incubation period, optical density (OD) was measured at 450 nm using a microplate reader (Biotek, Elx800, USA). The number of viable cells was calculated using the following formula:

(1) $$\text{Cell viability}\;\left(\%\right)=\frac{\mathrm{OD}\;\text{value of sample}}{\mathrm{OD}\;\text{value of control}}*100$$

#### 2.7.3 In Vitro Antidiabetic Activity

To evaluate the in vitro antidiabetic activities of PBE, α‐amylase and α‐glucosidase enzyme inhibition assays were conducted. Acarbose, a commercially available drug used in the treatment of Type II diabetes, served as the reference inhibitor, while phosphate buffer was used as the substrate (Kutlu 2024). PBE solutions prepared at different concentrations (0.0156, 0.0313, 0.0625, 0.1250, 0.2500, 0.3750, 0.5000, 0.7500, and 1.0000 mg/mL) were combined with 100 μL of 0.01 M sodium phosphate buffer (pH 6.9) and 100 μL of enzyme solution (α‐glucosidase: 1 U/mL/min; α‐amylase: 4.5 U/mL/min). The mixtures were preincubated at 25°C for 10 min. For the α‐glucosidase assay, 100 μL of 4‐nitrophenyl α‐D‐glucopyranoside (NPG) solution (5 mM) was added to the reaction mixture, which was then incubated for an additional 30 min at 25°C. The reaction was terminated by adding 2 mL of 0.1 M Na_2_CO_3_. Then the absorbance was measured at 405 nm after incubation. For the α‐amylase assay, 100 μL of 1% starch solution was added and incubated for another 30 min at 25°C. The reaction was stopped by placing the tubes in a boiling water bath for 5 min, after cooling to the room temperature. The absorbance was measured at 540 nm. In both assays, control samples were prepared by replacing the sample with buffer solution, and enzyme inhibition was calculated accordingly.

#### 2.7.4 In Vitro Acetylcholinesterase (AChE) and Butyrylcholinesterase (BChE) Inhibitory Activities

In this analysis, AChE from electric eel and BChE from horse serum were used as enzyme sources. For the assay, 20 μL of the sample solution (diluted from a 50 mg/mL DMSO stock solution) was mixed with 140 μL of phosphate buffer (0.1 M, pH 6.8) and 20 μL of AChE (5 × 10^**−**3^ M) or BChE (5 × 10^**−**3^ M) enzyme solution. The mixture was incubated for approximately 10 min. The reaction was initiated by adding 10 μL of 3 mM DTNB, followed by 10 μL of 0.71 mM acetylthiocholine iodide (AChI) for AChE or 0.2 mM butyrylthiocholine iodide (BChI) for BChE. The inhibitory activity of the PBE on AChE and BChE was measured at 405 nm using a microplate reader (Epoch, USA), following the method outlined by Bozkurt et al. (2021) and Kutlu (2024).

### 2.8 Statistical Evaluation

In the study, all measurements were conducted in duplicate with three parallels, and the results were expressed as mean ± standard deviation. Statistical analyses were performed using SPSS software (IBM Statistics, version 20, USA). Differences between samples were evaluated using one‐way analysis of variance (ANOVA), and Tukey's multiple comparison test was applied to determine the significance of differences between the parameters of the tested samples (*p* < 0.05).

## 3 Results and Discussion

### 3.1 Extraction Yield (EY) of PBE

The extraction yields (EY) of PBE are presented in Table 1, with values ranging from 7.03% to 15.03% under varying extraction conditions. As shown in Table 1, the highest EY was obtained at 30°C, 6 min, and 100 W (R5), while the lowest EY was recorded at 20°C, 3 min, and 100 W (R6). For comparison, EY values reported for other bark extracts include 6.10% for *Larix decidua* bark extracts obtained via ultrasound‐assisted extraction (Sillero et al. 2021), 10.14% for MeOH:H_2_O extracts of *Quercus suber* L. cork (Touati et al. 2015), and 6.39% for ethanol‐water extracts of *Quercus faginea* bark (Ferreira et al. 2018). These variations in EYs could be attributed to several factors such as the type of raw material, biological tissue type, pretreatment methods applied to the tissue prior to extraction, and the localization of bioactive components within tissue structures. Additionally, parameters such as moisture content and particle size also play a critical role in EY (Vilkhu et al. 2008).

**FIGURE 1:**
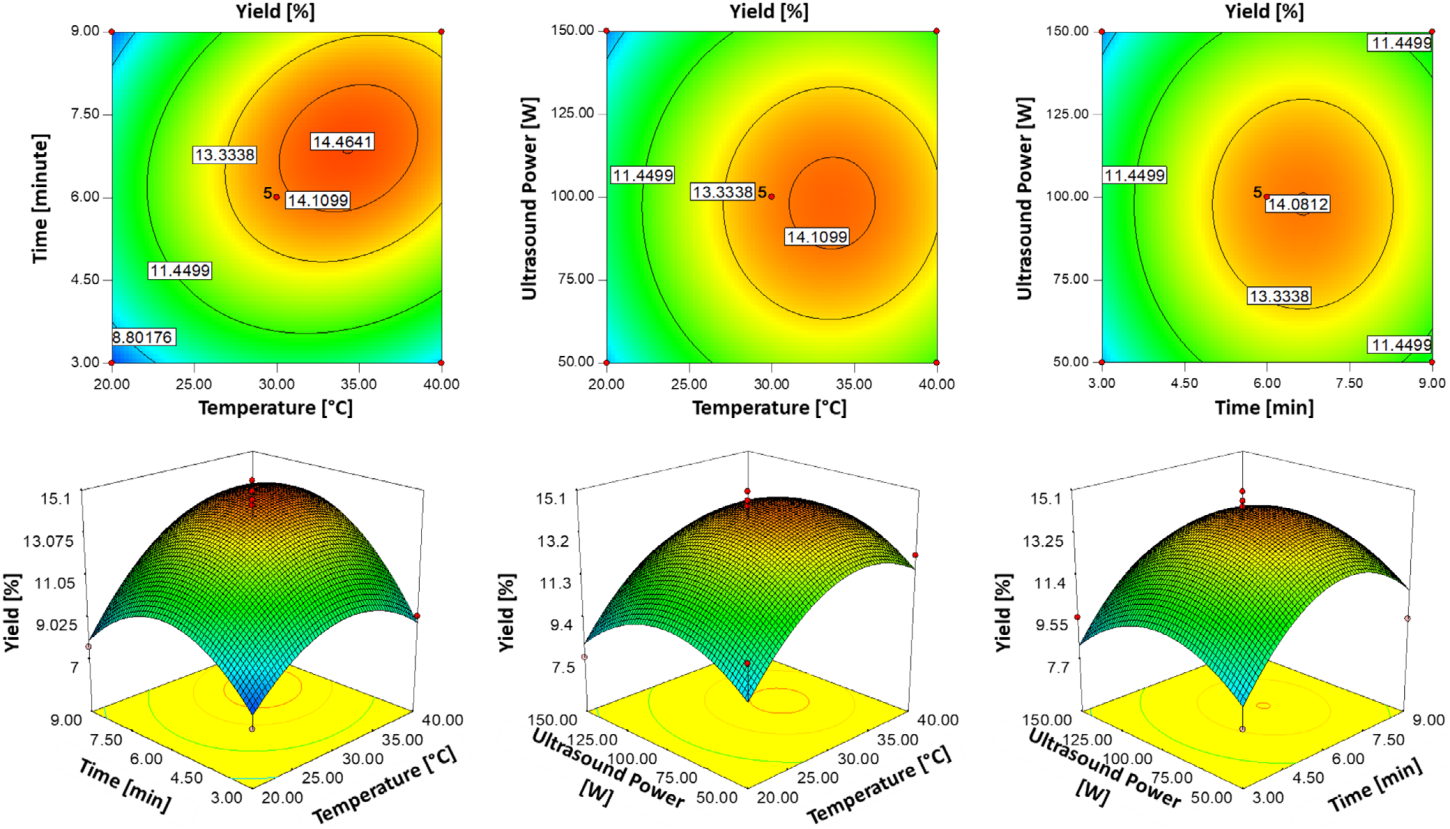
Three‐dimensional response surface and contour plots illustrating the effects of temperature (°C), time (min) and ultrasonic power (W) on the extraction yield percentage.

Insignificant variables were excluded from the regression model to refine the analysis. The regression correlation efficiency (*R* ^2^ = 0.8588, Table 2) indicated that the model could explain 85.88% of the variability in the data. The model's significance was confirmed by an *F*‐value of 4.73 and a *p*‐value below 0.05, demonstrating the adequacy of the quadratic model for representing the experimental results. Analysis of variance highlighted that the terms *X*_1_ (*p* ≤ 0.05), *X*_1_^2^ (*p* ≤ 0.05), *X*_2_^2^ (*p* ≤ 0.05) and *X*_3_^2^ (*p* ≤ 0.05) were statistically significant. In the equation predicting the EY of PBE (Table 2), the linear term for extraction temperature (*X*_1_) exhibited a positive coefficient, indicating that increased temperatures contributed positively to extraction yield. This factor had the highest coefficient value among all variables, emphasizing its dominant influence on the extraction process. The effects of independent variables on EY were visualized through two‐dimensional (2D) contour plots and three‐dimensional (3D) surface plots (Figure 1). These plots explored the interactions between key parameters: extraction temperature and time, extraction temperature and ultrasonic power, and extraction time and ultrasonic power. The results revealed that the highest extraction yield (14.46%) was achieved at approximately 34°C, with an ultrasonic power of 100 W and an extraction time of 7 min. Deviations from these optimal conditions led to a noticeable decline in extraction efficiency. Temperature was identified as the most critical parameter influencing EY. The EY increased with rising temperatures up to ~34°C, as higher temperatures facilitated the swelling of plant tissues, enhanced solvent penetration, and promoted the release of bioactive components. This phenomenon aligns with findings by Ezzati et al. (2020), who reported that while moderate heat improved extraction by loosening cell structures, excessive heat led to structural breakdown and diminished efficiency. Extraction time also played a crucial role. The EY increased steadily until 7 min, after which prolonged exposure led to a decline. Ezzati et al. (2020) attributed this pattern to the initial swelling of plant materials and enhanced solvent infusion, followed by extract degradation and fragmentation during extended ultrasound exposure.

**TABLE 2:** Predicted model equations and regression coefficients value for black pine bark extracts.

Parameters	Predicted model equations	*R* ^2^	*R* ^2^ adj	CV (%)	Adequate precision
Yield (%)	*Y* = −22.77000 + 1.21363*X_1_−0.021163*X_1_^2^−0.28097*X_2_^2^–7.50500E‐004*X_3_^2^	0.8588	0.7772	4.07	5.36
TPC (mg GAE/g dbe)	*Y* = −243.16000 + 14.04843*X_1_ + 12.45775*X_2_ + 1.74929*X_3_−0.20449*X_1_^2^−1.37603*X_2_^2^−8.23470E‐003 * *X*_3_^2^	0,9738	0,9400	4.07	17.67
TCT (mg CE/g dbe)	*Y* = −28.39625 + 2.28560*X_1_ + 0.60633*X_2_−0.032098*X_1_^2^−7.57900E‐004*X_3_^2^	0.8965	0.7633	8.16	8.65
FRAP activity (mg TEAC/g dbe)	*Y* = −192.95250 + 31.61778*X_1_−0.51035*X_1_^2^−3.24003*X_2_^2^−0.023465*X_3_^2^	0.9689	0.9290	2.15	14.39
ABTS activity (mg TEAC/g dbe)	*Y* = −643.52125 + 65.18070*X_1_ + 12.98808*X_2_ + 4.90748*X_3_−0.97101*X_1_^2^−0.020895*X_3_^2^	0.9026	0.7774	5.56	9.06

Abbreviations: CE, catechin equivalents; dbe, dried bark extract; FRAP, ferric reducing antioxidant power; GAE, gallic acid equivalents; TCT, total condensed tannin; TEAC, Trolox equivalents antioxidant capacity; TPC, total phenolic content; *X*_1_, extraction temperature; *X*_2_, extraction time; *X*_3_, ultrasonic power.

### 3.2 Effects of Independent Factors on TPC

Table 1 presents the TPC values of PBE, which ranged between 62.76 and 127.65 mg GAE/g dbe under different extraction conditions. The maximum TPC value was obtained at 30°C, 6 min, and an ultrasonic power of 100 W (R14), exceeding the TPC recorded at 20°C, 6 min, and 50 W (R12). For comparison, varying TPC values were reported for water extracts of *Quercus faginea* bark (630.33 mg GAE/g extract) (Ferreira et al. 2018), ethanol extracts of *Picea abies* bark (851 mg GAE/g extract) (Neiva et al. 2018), and MeOH:H_2_O extracts of *Quercus suber* L. cork (786.97 mg GAE/g extract) (Touati et al. 2015). These variations highlighted the influence of extraction parameters, plant species, extraction solvent type, and raw material types on TPC values.

Table 2 provides the polynomial equation for TPC findings, outlining the significant factors and *R* ^2^ value. Nonsignificant variables were excluded to improve the model. The regression coefficient (*R* ^2^ = 0.9738) indicates that the model accurately predicts TPC values based on extraction parameters, including temperature, time, and ultrasonic power. The *F*‐value of 56.87 and a *p*‐value below 0.01 confirm the model's robustness and suitability for the experimental data. Furthermore, the lack of fit test revealed no significant effects (*p* > 0.05), further supporting the adequacy of the model. Analysis of variance identified the linear term for *X*_1_ (*p* ≤ 0.01), *X*_2_ (*p* ≤ 0.05), and *X*_3_ (*p* ≤ 0.05) as well as quadratic terms (*X*_1_^2^ (*p* ≤ 0.01), *X*_2_^2^ (*p* ≤ 0.01) and *X*_3_^2^ (*p* ≤ 0.01)) as significant contributors to the TPC. However, the interaction terms were not statistically significant (*p* > 0.05), indicating that while individual parameters had a significant impact, their combined effects did not exhibit notable interactions within the tested ranges.

The response surface analysis yielded a polynomial equation that highlighted the influence of extraction parameters on TPC. The analysis revealed that TPC values increased with rising extraction temperature (*X*_1_), as evidenced by its positive coefficient, which also had the highest value, indicating the most significant impact among the variables. Similarly, positive coefficients for extraction time (*X*_2_) and ultrasonic power (*X*_3_) suggested that these factors also contributed to the overall increase in TPC values. The observed enhancement in phenolic extraction at elevated temperatures aligns with previous studies reporting that high temperatures promoted phenolic solubility by enhancing solvent penetration into the plant matrix (Belwal et al. 2016). Conversely, the squared terms exerted a notable negative effect on TPC, with *X*_2_^2^ showing the most significant influence, followed by *X*_1_^2^ and *X*_3_^2^. While *X*_1_ positively influenced TPC, the negative quadratic effect of *X*_1_^2^ indicated the presence of an optimal temperature, beyond which TPC values began to decline. Similarly, an extended extraction time positively impacted TPC values, consistent with findings by Dranca and Oroian (2016), which suggested that prolonged extraction facilitates phenolic release. However, excessive time or temperature could lead to degradation or loss of phenolic compounds.

**FIGURE 2:**
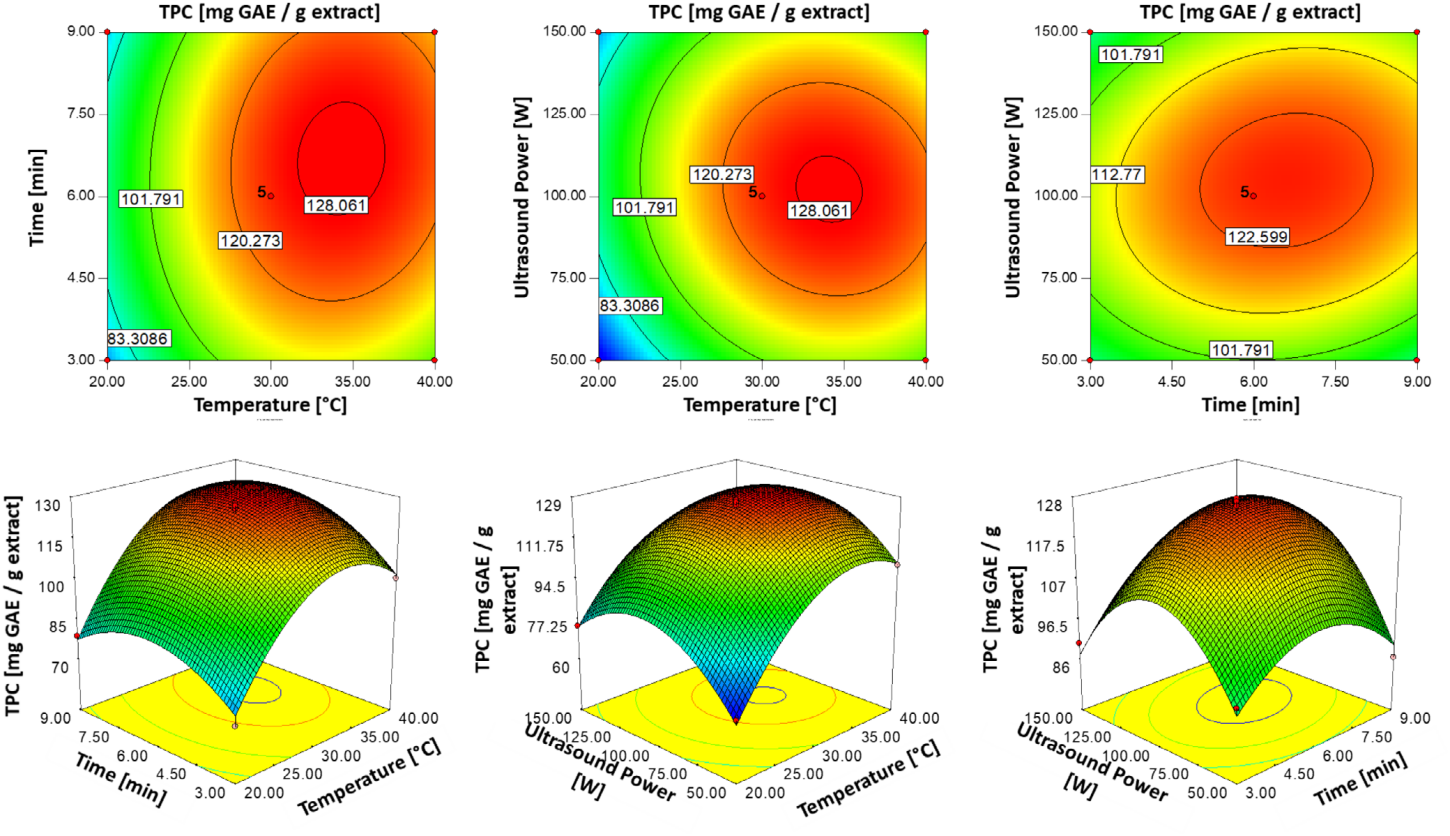
Three‐dimensional response surface and contour plots illustrating the effects of temperature (°C), time (min) and ultrasonic power (W) on TPC values (mg GAE/g dbe).

Figure 2 illustrates the interactions between extraction parameters, such as extraction temperature and time (*X*_1_ *X*_2_), temperature and ultrasonic power (*X*_1_ *X*_3_), and time and ultrasonic power (*X*_2_ *X*_3_). The response surface plots pinpointed the optimal conditions for maximizing TPC in PBE: approximately 34°C, 7 min, and 100 W ultrasonic power. Deviations from these optimal settings resulted in decreased TPC values, emphasizing the critical role of parameter optimization. These findings underscore the importance of balancing the extraction parameters to maximize TPC while minimizing potential losses due to adverse conditions such as increased temperatures or prolonged extraction periods.

### 3.3 Effects of Independent Factors on TCT

The TCT values of PBE ranged from 12.49 to 22.35 mg CE/g dbe, with the highest TCT observed in sample R4 (22.35 mg CE/g dbe) under extraction conditions of 30°C, 9 min, and 150 W ultrasonic power (Table 1). Conversely, the lowest TCT was recorded in sample R6 (12.49 mg CE/g of dbe) at 20°C, 3 min, and 100 W ultrasonic power. Comparative studies have reported TCT values for ethanol extracts of *Picea abies* bark (360 mg CE/g extract) (Neiva et al. 2018), water extracts of the *Picea abies* bark samples (40 mg CE/g extract) (Sillero et al. 2021), EtOH:H_2_O extracts of *Quercus faginea* bark (220.74 mg CE/g extract) (Ferreira et al. 2018), MeOH:H_2_O extracts of *Quercus suber* L. cork (29.54 mg CE/g extract) (Touati et al. 2015).

The *R* ^*2*^ and the estimated equation for the TCT value (significant terms were noted) were presented in Table 2. The *R* ^*2*^ value for TCT was 0.8965, indicating the satisfactory predictability of values based on extraction time, ultrasonic power, and extraction temperature. The model's *F*‐value was 6.73, with a statistically significant *p*‐value (*p* ≤ 0.01).

The lack of fit test confirmed that the model fit well with the data, as the *p*‐value exceeded 0.05, indicating no significant lack of fit at the 95% confidence level. Analysis of variance identified extraction temperature (*X*_1_, *p* ≤ 0.01), extraction time (*X*_2_, *p* ≤ 0.05), extraction temperature squared (*X*_1_^2^, *p* ≤ 0.01), and ultrasonic power squared (*X*_3_^2^, *p* ≤ 0.05) as significant factors influencing TCT values.

**FIGURE 3:**
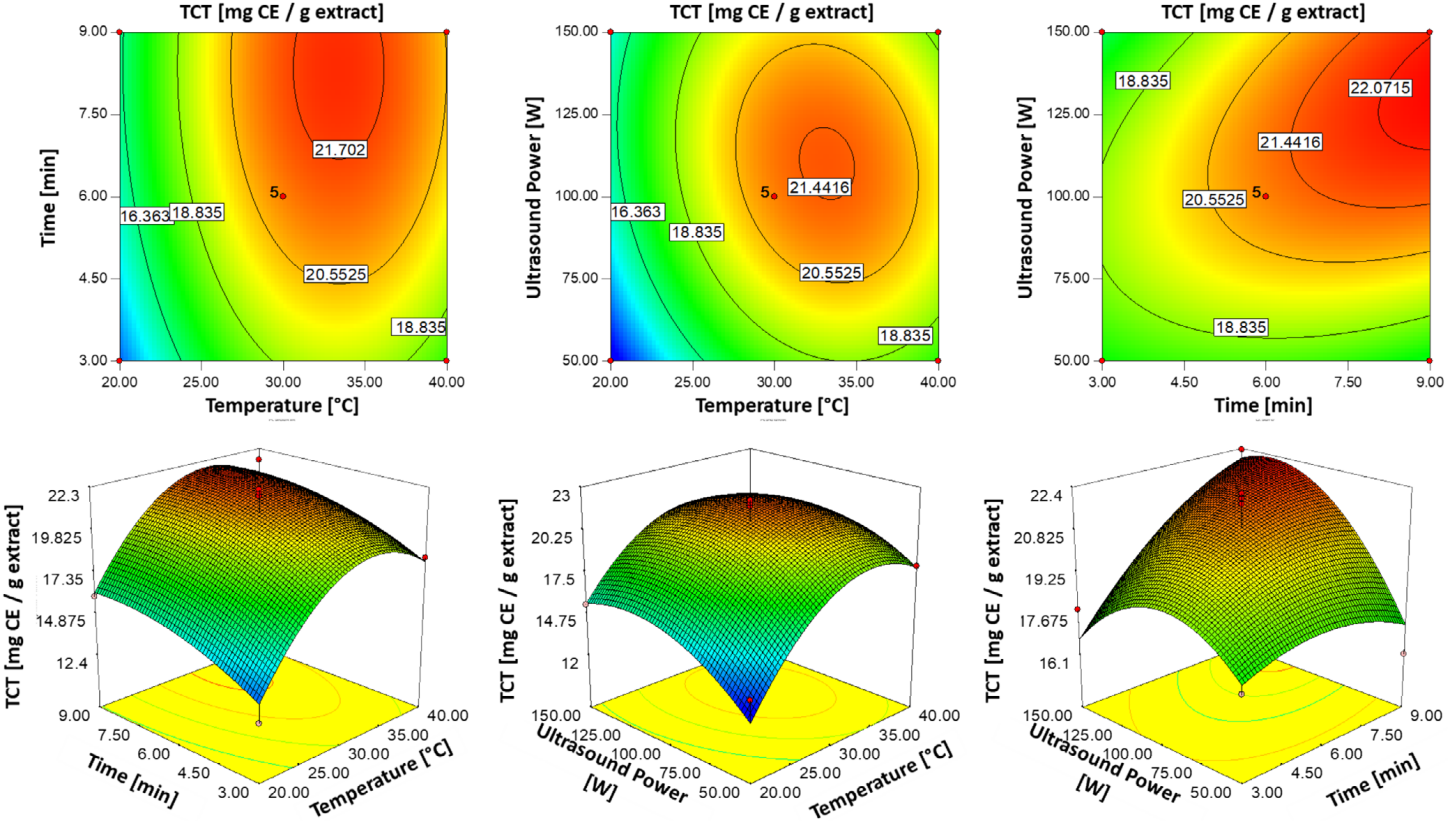
Three‐dimensional response surface and contour plots illustrating the effects of temperature (°C), time (min) and ultrasonic power (W) on TCT values (mg CE/g dbe).

Among these variables, extraction temperature (*X*_1_) had the most substantial positive effect on TCT, while the quadratic term (*X*_1_^2^) exhibited the strongest negative influence, followed by ultrasonic power squared (*X*_3_^2^). These effects were visualized in Figure 3 through 2D contour plots and 3D surface plots, illustrating the relationships between TCT values and the interaction of extraction parameters including extraction temperature and extraction time and ultrasonic power. The plots revealed that TCT reached its maximum around 34°C, regardless of extraction time, highlighting the critical role of temperature optimization. Prolonged extraction time also positively influenced TCT, with the peak value observed at 9 min. Additionally, when temperature was held constant, the maximum TCT was achieved at an ultrasonic power of approximately 135 W. These findings emphasized the importance of optimizing extraction parameters, particularly temperature and time, to maximize the TCT while avoiding conditions that could lead to reduced efficiency.

### 3.4 Effects of Independent Factors on FRAP

The FRAP values of PBE varied from 520.76 to 680.75 mg TEAC/g dbe, as outlined in Table 1. The highest FRAP value was observed under extraction conditions of 30°C, 6 min, and 100 W (R14), while the lowest value occurred at 20°C, 6 min, and 50 W (R12). Comparisons with related studies reported FRAP values for ethanol extracts of *Picea abies* bark (10 mmol Fe^2+^/g extract) (Neiva et al. 2018), water extracts of *Picea abies* bark (2.6 mmol Fe^2+^/g extract) (Ezzati et al. 2020), ethanol‐water extracts of *Quercus faginea* bark (4.44 mM TEAC/g extract) (Ferreira et al. 2018), and ethanol extracts of *Acacia dealbata* bark (412 ± 10 TE/g) (Abilleira et al. 2021). Lower values were reported for *Pinus pinaster* bark ethanolic extracts (145 ± 2 TE/g) and water extracts (126 ± 3 TE/g) (Abilleira et al. 2021).

The quadratic regression model for FRAP values showed an *R* ^2^ value of 0.9689 (Table 2), reflecting excellent predictive reliability. The *F*‐value of 24.24 was statistically significant at the 99% confidence level, while the lack of fit test (*p* > 0.05) indicated no significant lack of fit. Analysis of variance revealed that extraction temperature (*X*_1_, *p* ≤ 0.01), as well as the squared terms for extraction temperature (*X*_1_^2^, *p* ≤ 0.01), squared terms for extraction time (*X*_2_^2^, *p* ≤ 0.01), and squared terms for ultrasonic power (*X*_3_^2^, *p* ≤ 0.01), were significant factors influencing FRAP activity.

The regression analysis demonstrated that extraction temperature (*X*_1_) had a strong positive effect on FRAP values, as indicated by its substantial coefficient (31.6178). Conversely, the negative coefficients of *X*_1_^2^, *X*_2_^2^, and *X*_3_^2^ suggested that excessively high levels of these parameters may antagonize FRAP activity, likely due to degradation processes.

**FIGURE 4:**
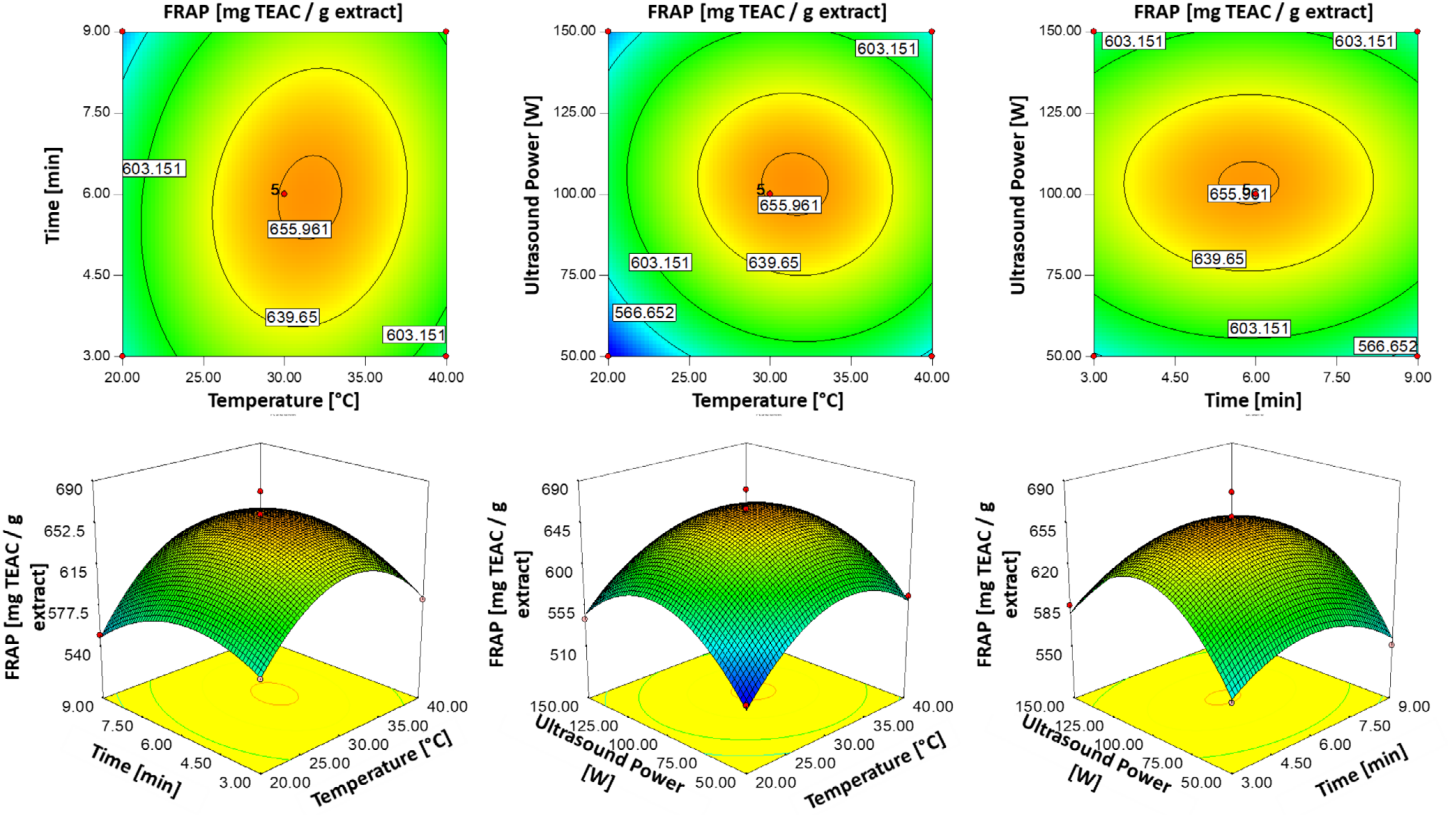
Three‐dimensional response surface and contour plots illustrating the effects of temperature (°C), time (min) and ultrasonic power (W) on FRAP values (mg TEAC/g dbe).

Figure 4 illustrates the effects of extraction parameters on FRAP values through 2D contour plots and 3D response surface diagrams. The optimal conditions for maximizing FRAP values were identified as an extraction temperature of approximately 31°C, a duration of 6 min, and ultrasonic power around 100 W. However, extending the extraction duration beyond optimal levels may lead to oxidative degradation of phenolic compounds, as supported by Chew et al. (2021), which reduced the antioxidant potential of the extracts. These findings underscored the importance of carefully balancing extraction parameters to maximize FRAP while minimizing oxidative losses during the extraction process.

### 3.5 Effects of Independent Factors on ABTS Antioxidant Activity

The ABTS activity values of PBE ranged from 540.81 to 830.46 mg TEAC/g dbe, as shown in Table 1. The highest ABTS activity was observed under extraction conditions of 30°C, 6 min, and 100 W (R7), while the lowest value occurred at 20°C, 6 min, and 50 W (R12). The model equation and the *R* ^2^ value were presented in Table 2. The quadratic response surface model yielded an *R* ^2^ value of 0.9026, CV% of 5.56, and a lack of fit test *p*‐value of 0.9312, indicating no significant lack of fit and good alignment between the model and the experimental ABTS activity values. The regression model was significant, as reflected by an *F*‐value of 7.21 (*p* ≤ 0.01).

The analysis of variance highlighted significant factors affecting ABTS activity, including *X*_1_ (*p* ≤ 0.01), *X*_2_ (*p* ≤ 0.05), *X*_3_ (*p* ≤ 0.05), *X*_1_^2^ (*p* ≤ 0.01), and *X*_3_^2^ (*p* ≤ 0.05). The quadratic model indicated that extraction temperature (*X*_1_) had the most substantial positive influence on ABTS activity values, followed by extraction time (*X*_2_) and ultrasonic power (*X*_3_). However, the quadratic terms *X*_1_^2^ and *X*_3_^2^ exhibited negative coefficients, suggesting diminishing returns or antagonistic effects at higher parameter values.

**FIGURE 5:**
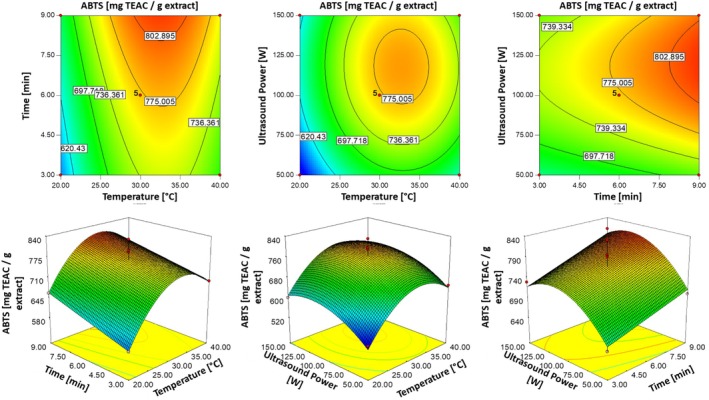
Three‐dimensional response surface and contour plots illustrating the effects of temperature (°C), time (min) and ultrasonic power (W) on ABTS values (mg TEAC/g dbe).

Figure 5 displays 2D contour plots and 3D response surface plots, illustrating the effects of extraction parameters on ABTS activity. The highest ABTS activity was achieved at an extraction temperature of approximately 33°C, regardless of extraction time. Additionally, increasing extraction time positively influenced ABTS values, with the peak activity observed at 9 min. The optimal ultrasonic power for maximizing ABTS activity was around 120 W.

Although both FRAP and ABTS assays assess antioxidant activity, the results did not exhibit a linear correlation. These variations could be arisen from the interaction of antioxidants with other compounds, as suggested by Moure et al. (2001). Moreover, distinct antioxidant mechanisms and variations in the relative antioxidant potential of individual model compounds may lead to variations between the test results. These findings also emphasized the complementary nature of FRAP and ABTS methods, highlighting the complexity of antioxidant interactions and the importance of employing multiple assays to obtain a comprehensive evaluation of antioxidant capacity.

### 3.6 Multiple Response Optimization

Multiple response optimization using the desirability function was performed to determine the optimum conditions for maximizing both extraction yield and bioactive properties of PBE, including TPC, TCT, FRAP, and ABTS activities. The analysis identified that the maximum extraction yield of 14.47% could be achieved with an extraction time of 6.89 min, an extraction temperature of 34.33°C, and an ultrasonic power of 98.45 W. Similarly, the predicted optimal conditions for maximizing the bioactive properties were established. The maximum values were estimated as 128.00 mg GAE/g dbe for TPC, 22.08 mg CE/g dbe for TCT, 802.04 mg TEAC/g dbe for ABTS activity, and 649.49 mg TEAC/g dbe for FRAP activity (Table 3). These values were achieved at an extraction time of 7.43 min, an extraction temperature of 32.52°C, and an ultrasonic power of 110.68 W.

**TABLE 3:** Validation of *Pinus nigra* bark extraction model.

Parametreler	Estimated maximum levels	Measured real levels
Yield (%)	14.47^a^	13.56 ± 0.31^a^
TPC (mg GAE/g dbe)	128.00^a^	126.15 ± 5.70^a^
TCT (mg CE/g dbe)	22.08^a^	21.43 ± 0.52^a^
FRAP (mg TEAC/g dbe)	649.49^a^	619.06 ± 29.53^a^
ABTS (mg TEAC/g dbe)	802.04^a^	750.77 ± 36.83^a^

*Note:* The same letters between the rows indicate that the differences are not statistically significant at the 0.05 level.

Abbreviations: CE: catechin equivalents; FRAP: ferric reducing antioxidant power; GAE: gallic acid equivalents; TCT: total condensed tannin; TEAC: trolox equivalents antioxidant capacity; TPC: total phenolic content.

The desirability function, which ranges from 0 to 1 (with 1 representing ideal conditions), demonstrated values exceeding 0.917 for all parameters. This indicates that the identified conditions provided a highly desirable balance between yield and bioactive potential. As the extraction efficiency deviated from the identified parameter values, a noticeable decrease is observed. Based on the findings, one of the key factors influencing the efficiency of PBE extraction has been identified as the extraction temperature. An increase in extraction temperature promotes higher efficiency up to a certain threshold, beyond which a significant decline in yield is observed. This approach highlighted the importance of optimizing extraction conditions to balance efficiency and the preservation of bioactive compounds, ensuring high‐quality extracts when minimizing thermal degradation. These optimized conditions highlight an effective and efficient strategy for maximizing the extraction of bioactive compounds from PBE, offering valuable insights for future applications.

### 3.7 LC–MS/MS

Qualitative and quantitative analyses of PBE identified 12 phenolic compounds and 3 organic acids, as detailed in Table 4. The content of these compounds, arranged in descending order, is as follows: succinic acid (16.35 mg/100 g) > oxalic acid (6.81 mg/100 g) > gentisic acid (7.58 mg/100 g) > gallic acid (5.90 mg/100 g) > 4‐hydroxybenzoic acid (5.80 mg/100 g) > vanillic acid (2.20 mg/100 g) > chlorogenic acid (1.51 mg/100 g) > caffeic acid (0.86 mg/100 g) > tartaric acid (0.43 mg/100 g) = sinapic acid (0.43 mg/100 g) = ferulic acid (0.43 mg/100 g) > coumaric acid (0.09 mg/100 g) = 3′‐hydroxycinnamic acid (0.09 ppm) > syringic acid (0.04 mg/100 g) > cinnamic acid (0.02 mg/100 g). The presence of 4‐hydroxybenzoic acid in PBE aligns with findings from a previous study on *Pinus nigra* bark, which identified phenolic constituents such as 4‐hydroxybenzaldehyde, vanillin, 4‐hydroxybenzoic acid, and 3,4‐dihydroxybenzaldehyde using GC–MS (Hafızoğlu et al. 2002). However, a notable difference is the detection of gallic acid in our study, which was absent in *Pinus nigra* bark. Additionally, the earlier research provided only qualitative identification of phenolic compounds without quantification, whereas our study offers a comprehensive quantitative profile. These findings highlighted the rich diversity of phenolic and organic compounds in PBE, underscoring its potential as a source of bioactive compounds. The variation in phenolic profiles between this study and previous research could be attributed to differences in species, extraction techniques, or analytical methods. This comprehensive identification and quantification provide valuable insights into the chemical composition of PBE, supporting its potential application in food, nutraceutical and pharmaceutical formulations.

**TABLE 4:** Phenolic compounds specified and quantified in PBE (mg/100 g) by LC–MS/MS.

Phenolic & organic compounds	Rt	Formula	MS^1^	MS^2^	∆ Rt	Fr	CE	CEV	*p*	Results
	(min)	F	[M‐H]^−^	[M‐H]^−^	(min)	(V)	(V)	(V)		(mg/100 g)
			(m/z)	(m/z)						
4‐Hydroxybenzoic acid	13.19	C_7_H_6_O_3_	137	65	2.70	100	28	4	Negative	5.80 ± 0.01
Caffeic acid	14.61	C_9_H_8_O_4_	179	135	3.80	80	22	4	Negative	0.86 ± 0.02
Chlorogenic acid	13.20	C_16_H_18_O_9_	353	191	3.30	180	26	4	Negative	1.51 ± 0.01
Cinnamic acid	23.18	C_9_H_8_O_2_	147	77	2.32	40	20	4	Negative	0.02 ± 0.01
Ferulic acid	18.17	C_10_H_10_O_4_	193	178	1.93	60	6	4	Negative	0.43 ± 0.01
Gallic acid	6.82	C_7_H_6_O_5_	169	79	3.64	40	22	4	Negative	5.90 ± 0.02
Gentisic acid	10.69	C_7_H_6_O_4_	153	108	3.81	70	18	4	Negative	7.58 ± 0.07
Coumaric acid	17.29	C_9_H_8_O_3_	163	119	2.92	60	8	4	Negative	0.09 ± 0.01
Oxalic acid	20.00	C_2_H_2_O_4_	203	131	5.00	180	22	4	Negative	6.81 ± 0.01
Sinapic acid	18.16	C_11_H_12_O_5_	223	93	1.82	100	34	4	Negative	0.43 ± 0.01
Succinic acid	34.46	C_4_H_6_O_4_	141	59	3.45	50	4	4	Negative	16.35 ± 0.01
Syringic acid	15.69	C_9_H_10_O_5_	197	182	2.70	70	10	4	Negative	0.04 ± 0.01
3′‐Hydroxycinnamic acid	17.29	C_9_H_8_O_3_	163	91	2.92	110	26	4	Negative	0.09 ± 0.01
Tartaric acid	10.00	H_2_C_4_H_4_O_6_	149	87	10.00	60	6	4	Negative	0.43 ± 0.01
Vanillic acid	14.89	C_8_H_8_O_4_	167	123	2.17	40	4	4	Negative	2.20 ± 0.02

Abbreviations: ∆Rt, delta retention time; CE, collision energy; CEV, cell accelerator voltage; CV, capillary voltage; F, formula; Fr, fragmentor; MS^1^, precursor ion; MS^2^, product ion; P, polarity; R_t_, retention time.

### 3.8 Antimicrobial Activity of PBE

The MIC of PBE obtained under optimized conditions was determined using the microdilution method in Nutrient Broth, with the results summarized in Table 5. The antibacterial activity of PBE was evaluated against two Gram‐negative bacteria ( *E. coli* and S. Typhimurium) and three Gram‐positive bacteria ( *L. monocytogenes* , *S. aureus* , and *B. cereus* ) due to their significant roles in foodborne diseases (Erol and Kutlu 2024b; Ferreira‐Santos et al. 2020). The findings demonstrated that PBE exhibited notable inhibitory effects on the tested bacterial strains, with MIC values ranging from 0.312 to 0.625 mg/mL. The MIC trend was as follows: *S. aureus* (0.312 mg/mL) = S. Typhimurium (0.312 mg/mL) < *B. cereus* = *E. coli* = *L. monocytogenes* (0.625 mg/mL). These results highlight the antibacterial efficacy of PBE, particularly against *S. aureus* and S. Typhimurium.

**TABLE 5:** Antimicrobial activities of *Pinus nigra* bark extract against various Gram‐positive and Gram‐negative bacteria.

	Microorganisms	MIC (mg/mL)
Gram+	*S. aureus* *(ATCC25923)*	0.312 ± 0.01
	*B. cereus* (FMC‐19)	0.625 ± 0.02
	*L. monocytogenes* (ATCC19118)	0.625 ± 0.01
Gram−	S. Typhimurium (ATCC14028)	0.312 ± 0.01
	*E. coli* (ATCC33150)	0.625 ± 0.02

Abbreviation: MIC, the minimum inhibitory concentration.

Comparative studies provide additional context for the antibacterial potential of related extracts. For instance, Nisca et al. (2021) reported that ultrasound‐assisted extraction of *Pinus nigra* inhibited methicillin‐resistant *S. aureus* (ATCC 43300) and *S. aureus* (ATCC 25923) at a concentration of 6.25 mg/mL, although Gram‐negative bacteria showed significantly lower susceptibility. Similarly, Royer et al. (2013) found that polyphenolic bark extracts from Canadian forest species (*Acer rubrum, Pinus banksania, Betula alleghaniensis*, and *Picea mariana* ) were more effective against Gram‐positive bacterial strains such as *L. ivanovii* and *E. coli* . In contrast, studies on essential oils and extracts from *Pinaceae* species have shown varying results. Loizzo et al. (2008) found that essential oil from *Pinus brutia* exhibited limited activity against *S. aureus* (10 mm inhibition zone) but had a more pronounced effect on *E. coli* (52 mm inhibition zone). Furthermore, Eryilmaz et al. (2016) noted that etheric extracts of *Pinus nigra* showed minimal activity against *S. aureus* , while extracts of *Pinus brutia* had no effect on *S. aureus* or *E. coli* . The results of the current study, alongside findings from previous research, suggested that PBE could hold significant importance as an antibacterial agent. Variations in antibacterial efficacy among extracts and methods may be attributed to differences in extraction techniques, compound profiles, and bacterial strain susceptibility.

### 3.9 Anticancer Activity of PBE

**FIGURE 6:**
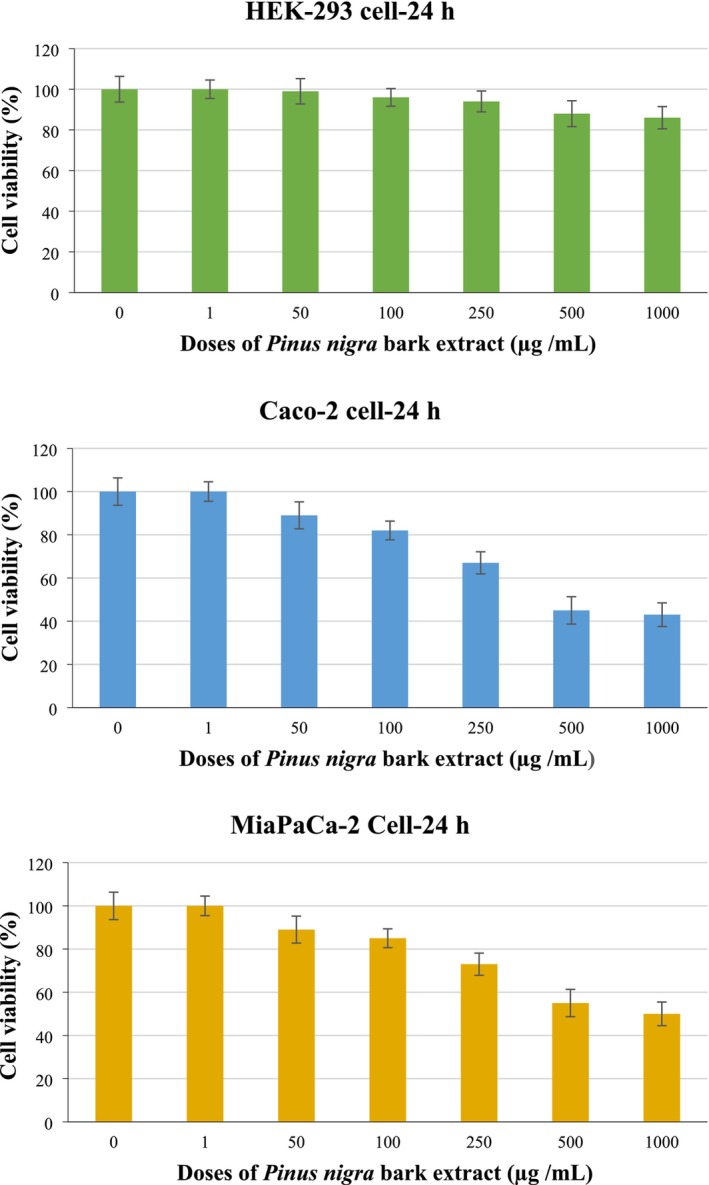
Cell viability percentages of Caco‐2, MIA PaCa‐2, and HEK‐293 cells depending on *Pinus nigra* bark extract concentration.

The in vitro anticarcinogenic activities of PBE were evaluated using the XTT assay on Caco‐2 and MIA PaCa‐2 cells, along with HEK293 cells. The results, as illustrated in Figure 6, indicated that PBE exhibited a weak cytotoxic effect on HEK293 cells, only at the highest dose of 1000 μg/mL, suggesting selective activity towards cancerous cells. In contrast, PBE demonstrated a dose‐dependent reduction in cell viability in Caco‐2 cells, with significant cytotoxic effects (*p* < 0.05) observed at concentrations above 100 μg/mL. A comparison of cell viability between Caco‐2 and HEK293 cells revealed significant differences across all experimental groups except at the 1 μg/mL and 10 μg/mL doses, highlighting the preferential cytotoxicity of PBE. Moreover, the IC_50_ values of PBE were determined as 696.10 μg/mL for Caco‐2 cells, 898.78 μg/mL for MIA PaCa‐2 cells, and 3313.67 μg/mL for HEK‐293 cells. These findings reveal that PBE exhibits a selective cytotoxic effect and shows higher activity against cancer cells (Caco‐2 and MIA PaCa‐2) compared to healthy HEK‐293 cells. Lower IC_50_ values are associated with greater cytotoxic potency, whereas higher IC_50_ values suggest reduced cytotoxicity. According to the screening criteria established by the National Cancer Institute (NCI), an extract is considered to have significant in vitro cytotoxic activity if its IC_50_ is below 30–40 μg/mL (Kutlu 2024; Erol and Kutlu 2024a; Erol and Kutlu 2024b). Therefore, the IC_50_ values observed for PBE in this study suggest that it does not meet the threshold for strong cytotoxic activity as per NCI standards.

The selected cancer cell lines, Caco‐2 and MIA PaCa‐2, exhibited moderate sensitivity to PBE, while the significantly higher IC_50_ value for HEK‐293 cells (human embryonic kidney) indicated lower cytotoxicity towards healthy cells. This selective cytotoxic profile suggested that PBE could have potential as a therapeutic agent with a preferable safety margin for healthy cells, though further refinement and investigation are necessary to enhance its efficacy. This aligns with previous studies employing similar cells to evaluate the potential anticancer properties of tree bark extracts (Mao et al. 2017). Several studies corroborated the anticancer potential of bark‐derived extracts. For instance, Tuli et al. (2021) reported strong anticancer activity for betulin, a compound derived from birch bark. Gascón et al. (2018) demonstrated the antiproliferative, apoptotic, and redox‐regulating properties of bark extracts from three pine species (*P. pinea, P. pinaster*, and *P. halepensis* ) on Caco‐2 cells, with *P. pinaster* bark extracts showing significant antioxidant and growth‐regulating effects on human melanoma cells (Touriño et al. 2005; Ferreira‐Santos et al. 2020). Additionally, Mao et al. (2017) found that bark extracts from *Pinus massoniana* suppressed the migration of lung cancer A549 cells, further highlighting the anticancer potential of bark‐derived bioactive compounds. These findings underscore the promise of PBE as a selective anticancer agent, particularly for gastrointestinal and pancreatic cancer cells, while sparing healthy cells at lower doses. Further studies to isolate and characterize the active compounds responsible for these effects could pave the way for developing novel cancer therapeutics.

### 3.10 In Vitro α‐Glycosidase/α‐Amylase‐Inhibitory Activity

The antidiabetic potential of PBE was assessed through its inhibitory activity against key enzymes involved in carbohydrate digestion: α‐glucosidase and α‐amylase. As shown in Table 6, PBE exhibited an IC_50_ value of 0.38 mg/mL against α‐glucosidase, outperforming the antidiabetic drug acarbose, which had an IC_50_ value of 0.72 mg/mL. Similarly, PBE showed an IC_50_ value of 0.46 mg/mL against α‐amylase, also surpassing acarbose, which recorded an IC_50_ value of 0.66 mg/mL. These findings highlighted PBE's notable antidiabetic activity, demonstrating a stronger inhibitory effect at lower concentrations compared to acarbose, a standard therapeutic agent used for managing type II diabetes.

**TABLE 6:** Anticholinesterase enzyme inhibition activities (%) at 2 mg/mL and IC_50_ values for antidiabetic enzyme inhibition activities of *Pinus nigra* bark extracts.

	Anticholinesterase activity		Antidiabetic activity	
	AChE (%)	BChE (%)	ɑ‐Glucosidase (mg/mL)	ɑ‐Amylase (mg/mL)
PBE	57.26 ± 0.23^b^	48.35 ± 0.21^b^	0.38 ± 0.02^b^	0.46 ± 0.02^b^
Galanthamine hydrobromide	90.45 ± 0.41^a^	86.48 ± 0.38^a^	—	—
Acarbose	—	—	0.72 ± 0.02^a^	0.66 ± 0.02^a^

Abbreviations: AchE, acetylcholinesterase; BchE, butyrylcholinesterase. Different letters between the columns demonstrate that the differences are statistically significant at the 0.05 level.

The inhibition of α‐glucosidase and α‐amylase enzymes plays a crucial role in managing postprandial glucose levels by delaying carbohydrate digestion and glucose absorption. This is particularly important for controlling postprandial hyperglycemia in type II diabetes. Moreover, PBE, which is rich in polyphenolic compounds, demonstrated a promising potential for antidiabetic effects through enzyme inhibition (Erol and Kutlu 2024a, 2024b). Diets rich in polyphenols have been shown to lower blood glucose levels by inhibiting the activity of key digestive enzymes (Kim et al. 2016). Some hydroxycinnamic acids, such as caffeic acid, chlorogenic acid, and ferulic acid, are known to exhibit significant hypoglycemic activity (Vinayagam et al. 2016). Specifically, chlorogenic acid has been reported to reduce diabetic complications like retinopathy by inhibiting retinal neoangiogenesis and improving sensory neural functions (Mei et al. 2018; Hong et al. 2017). Ferulic acid has been shown to lower blood glucose levels and enhance insulin secretion in diabetic animal models, providing further evidence for the potential of natural compounds in diabetes management (Jung et al. 2007; Kasetti et al. 2012).

Comparable results have been reported in the literature. For instance, Yesmin et al. (2019) documented that α‐amylase inhibitory activity of ethanolic extracts from *Michelia champaca* bark was 64.70% inhibition at a concentration of 1000 μg/mL, which was comparable to the standard drug acarbose (51.59%). Similarly, Ramachandran et al. (2013) reported the α‐amylase (10.14%, at 1 μg/mL) and α‐glucosidase (5.91%, at 25 μg/mL) inhibitory activities of *Terminalia paniculata* bark extracts, although these values were lower than those observed in the present study for PBE. The results suggest that PBE may serve as a promising natural alternative or complementary agent in diabetes treatment, offering effective enzyme inhibition with potential advantages in terms of natural bioactivity and reduced side effects. However, clinical trials are essential to confirm the efficacy and safety of PBE in vivo, further validating its potential as a therapeutic agent for diabetes management.

### 3.11 In Vitro AChE/BChE‐Inhibitory Activity

The anticholinesterase activity of PBE at a concentration of 2 mg/mL is summarized in Table 6, alongside the reference standard galantamine hydrobromide. The inhibition percentages for PBE were 57.26% for AChE and 48.35% for BChE, demonstrating moderate inhibitory effects. In comparison, the standard galantamine hydrobromide exhibited significantly higher inhibition percentages of 88.57% for AChE and 85.72% for BChE.

To our knowledge, this study represents the first experimental evaluation of the in vitro AChE/BChE inhibitory activities of PBE. For comparison, Ustun et al. (2012) investigated the anticholinesterase activities of *Pinus nigra* extracts of twings and needles of *Pinus nigra* obtained with different solvents. The AChE inhibition percentages of acetone, ethyl acetate, and methanol extracts from twigs were approximately 36%, 25%, and 22%, respectively, while the corresponding values for needles were 18%, 10%, and 19%, significantly lower than the activity observed for PBE. The BChE inhibitory activity of twig extracts was reported as approximately 38% (acetone), 23% (ethyl acetate), and 42% (methanol), whereas the needle extracts showed weaker inhibition at 2% (acetone), 9% (ethyl acetate), and 19% (methanol). Galantamine hydrobromide consistently exhibited superior inhibitory activity (~90%) across both enzymes.

In conclusion, while PBE demonstrated moderate AChE and BChE inhibition, its activity is considerably weaker than that of galantamine hydrobromide, a commercially available inhibitor with well‐established neuroprotective properties. These findings suggested that while PBE showed some potential, further investigations and optimizations would be required to evaluate its feasibility as a therapeutic agent targeting cholinesterase‐related disorders.

## 4 Conclusion

The study optimized the extraction conditions of PBE using the Box–Behnken Design (BBD) for maximization of the extraction yield and bioactive properties including TPC, TCT, and antioxidant activities (ABTS and FRAP). The optimal conditions were determined as an extraction temperature of 32.52°C, an extraction time of 7.43 min, and an ultrasonic power of 110.68 W. Under these conditions, the maximum values achieved were 128.00 mg GAE/g dbe for TPC, 22.08 mg CE/g dbe for TCT, 649.49 mg TEAC/g dbe for FRAP, and 802.04 mg TEAC/g dbe for ABTS. LC–MS/MS analysis identified 12 phenolic compounds and 3 organic acids, with succinic acid, oxalic acid, and genistic acid being the most abundant. Cytotoxicity evaluations revealed that PBE exhibited moderate effects against MIA PaCa‐2 and Caco‐2 cancer cells while showing minimal cytotoxicity towards the healthy HEK‐293 cells. Furthermore, PBE demonstrated potential for managing diabetes and Alzheimer's disease, as evidenced by its inhibitory activities against α‐amylase and α‐glucosidase. Although PBE showed moderate inhibition of AChE and BChE, its activity was significantly lower compared to galantamine hydrobromide, a commercially available inhibitor. Overall, these findings underscored the therapeutic potential of PBE as a rich source of bioactive compounds with diverse biological activities, offering promising applications in health and disease management.

## Author Contributions

**Kubra Feyza Erol:** conceptualization (equal), data curation (equal), formal analysis (equal), investigation (equal), methodology (equal), resources (equal), supervision (equal), validation (equal), visualization (equal), writing – original draft (equal). **Gozde Kutlu:** software (equal), visualization (equal), writing – original draft (lead), writing – review and editing (lead). **Necattin Cihat Icyer:** data curation (equal), writing – original draft (equal). **Fatih Tornuk:** conceptualization (lead), validation (lead), project administration (lead), supervision (lead), resources (lead), data curation (lead).

## Ethics Statement

The authors have nothing to report.

## Conflicts of Interest

The authors declare no conflicts of interest.

## Data Availability

Data available on request from the corresponding author.
